# Blood baseline neutrophil count predicts bevacizumab efficacy in glioblastoma

**DOI:** 10.18632/oncotarget.10898

**Published:** 2016-07-28

**Authors:** Aurélie Bertaut, Caroline Truntzer, Rachid Madkouri, Coureche Guillaume Kaderbhai, Valentin Derangère, Julie Vincent, Bruno Chauffert, Marie Hélene Aubriot-Lorton, Wahlid Farah, Klaus Luc Mourier, Romain Boidot, Francois Ghiringhelli

**Affiliations:** ^1^ Biostatistics unit Georges Francois Leclerc Cancer Center, Dijon, France; ^2^ CLIPP, Research Center, University of Burgundy, Dijon, France; ^3^ Department of Neurosurgery, CHU, Dijon, France; ^4^ Department of Medical Oncology, Georges Francois Leclerc Cancer Center, Dijon, France; ^5^ Platform of Transfer in Cancer Biology Genetic and histology, Georges Francois Leclerc Cancer Center, Dijon, France; ^6^ Department of Medical Oncology, University Hospital Amiens, Amiens, France; ^7^ Department of Pathology, CHU, Dijon, France; ^8^ INSERM U866, Dijon, France; ^9^ University of Bourgogne Franche-Comté, Dijon, France

**Keywords:** glioblastoma, bevacizumab, prognostic factor

## Abstract

Bevacizumab is used to treat glioblastoma; however, no current biomarker predicts its efficacy. We used an exploratory cohort of patients treated with the radiochemotherapy then bevacizumab or chemotherapy at recurrence (*N* = 265). Bevacizumab use increased median overall survival (OS) 18.7 *vs* 11.3 months, *p* = 0.0014). In multivariate analysis, age, initial surgery, neutrophil count, Karnofsky status >70% and bevacizumab administration were independent prognostic factors of survival. We found an interaction between bevacizumab use and baseline neutrophil count. The cut-off value for the neutrophil count was set at 6000/mm^3^. Only patients with a high neutrophil count benefited from the bevacizumab treatment (17.3 *vs* 8.8 months *p* < 0.0001). We validated this result using data from the TEMAVIR trial, which tested the efficacy of neoadjuvant bevacizumab plus irinotecan versus radiochemotherapy in the first-line treatment of glioblastoma. Transcriptomic data from TCGA underlined that *CSF3* expression, the gene encoding G-CSF, the growth factor for neutrophils, correlated with VEGF-A-dependent angiogenesis. In another independent cohort (BELOB trial), which compared lomustine versus lomustine plus bevacizumab at recurrence, bevacizumab only benefited patients with high *CSF3* expression in the tumor. These data suggest that only patients with a high peripheral neutrophil count before bevacizumab treatment benefited from this therapy.

## INTRODUCTION

Glioblastomas (GBM) are the most frequent brain tumors [1, 2]. The standard of care for newly diagnosed GBM entails surgical resection, followed by radiochemotherapy with concurrent temozolomide and then six months of adjuvant temozolomide [3]. Despite this aggressive therapy, the prognosis of GBM remains poor with median overall survival of only 14 months [3].

High-grade gliomas are highly vascularized tumors leading to the rational use of antiangiogenic agents to treat this disease [4–7]. Bevacizumab is a humanized monoclonal antibody raised against circulating VEGF-A. This drug is commonly used in combination with standard chemotherapy to treat metastatic colorectal, lung, ovarian, renal and breast carcinomas [8]. In GBM, some phase 2 trials have reported high response rates and high progression-free survival (PFS) with bevacizumab combined with chemotherapy [9–12]. Accordingly, bevacizumab is frequently used for recurrent GBM after radiotherapy. However, two phase III trials testing the effect of bevacizumab with concomitant radiochemotherapy [13, 14] failed to show any overall survival (OS) benefit. In addition, the EORTC 26101 trial, which tested the effect of bevacizumab with lomustine in recurrent GBM [15], failed to demonstrate that bevacizumab improved OS. Consequently, the use of bevacizumab remains controversial and some health care agencies (like in France) have not approved bevacizumab for GBM. Nonetheless, a biomarker to predict the efficacy of bevacizumab in GBM is an unmet need.

The neutrophil count and the neutrophil-to-lymphocyte ratio at baseline have been associated with GBM prognosis [16–19]. Preclinical reports suggest that neutrophils may promote tumor neoangiogenesis [20–22], thus raising the hypothesis that a high neutrophil count could be associated with a better response to anti-VEGF therapy. To address this question, we used two cohorts of GBM patients to determine whether the baseline neutrophil count could be a predictor of bevacizumab efficacy.

## MATERIALS AND METHODS

### Exploratory cohort

Two hundred and sixty-five patients were extracted from the Dijon Anti-cancer Center cohort, which registers all patients aged 16 years and older with a new diagnosis of histologically confirmed GBM and treated at the center with radiochemotherapy according to the Stupp Protocol3. The pathological diagnosis was also made by neuropathologists at Dijon Teaching Hospital in accordance with WHO guidelines [23]. In any cases of doubt about the pathological diagnosis, Dominique Figarella, the national reference for neuropathology, was asked to make the diagnosis. All patients provided written informed consent to undergo surgery and chemoradiotherapy. They were also asked to give written consent for their personal data to be used anonymously for research purposes. The study was approved by the local internal review board and the CNIL (Commission National Informatique et Liberté) DR-2014-556 and CCTIRS (Comité consultatif sur le traitement de l'information en matière de recherche) n° 14.702.

### TEMAVIR cohort

This cohort included 120 patients aged from 18 to 70 with unresectable glioblastoma, with a Karnofsky score higher than 50% recruited between April 2009 and January 2011. Patients were randomized into an experimental arm consisting of neoadjuvant intravenous bevacizumab and irinotecan every 2 weeks for four cycles before classical radiotherapy with concomitant temozolomide and bevacizumab every 2 weeks. Adjuvant bevacizumab and irinotecan were given every 2 weeks for 6 months. The control arm consisted of the classical Stupp regimen. The use of bevacizumab was allowed at progression in the control arm. The protocol and the results of the trial have been published previously [24].

### Statistical analysis

All patients were followed up until death or the date of the cut-off for study analysis. OS was calculated from the date when therapy started to the date of death. Median follow-up was calculated using the reverse Kaplan Meier method. OS probabilities were estimated using the Kaplan-Meier method and were compared by the log-rank test. The optimal cut-off was determined in an exploratory cohort using Cutoff Finder software [25]. The optimal cut off was defined as the point with the most significant (log-rank test) split. A multivariate Cox proportional hazard regression model was applied to assess independent prognostic effects for OS. The hazard ratios (HR) were given with their 95% confidence intervals (CI). All variables with a univariate Cox *p* value ≤ 0.20 were eligible for multivariate analyses. The multivariate model was adjusted for the use of neoadjuvant chemotherapy, sex, age, preoperative Karnofsky score (assessed the day before surgery), surgery (resection vs biopsy) and hemoglobin level. Neutrophil and lymphocyte counts were tested to be included in the survival model. Correlations between co-variables were first tested for eligible variables. To prevent collinearity, when two variables were significantly correlated, one variable was retained according to its clinical relevance or to the value of the likelihood ratio. The stability of hazard ratios was internally validated using bootstrapping (265 replications). Interactions between treatment with bevacizumab and neutrophil counts were tested in the whole population. All reported *p* values are two sided. The statistical significance level was set at *p* < 0.05. Analyses were performed using SAS 9.3 (Statistical Analysis System).

### Transcriptomic analysis

Gene expression analysis was performed using Rgui open-source software (http://cran.r-project.org) in 202 patients suffering from GBM whose tumors had been analyzed by gene expression array (Affymetrix) by the International Genomics Consortium. The data were downloaded from TCGA website (https://tcga-data.nci.nih.gov/docs/publications/gbm_exp/). Samples were selected according to the following criteria: 1) an average percentage of necrosis less than 40% on top and bottom slides; 2) microarray quality controls within standards and 3) high-quality data on each of the three gene expression platforms used. All specimens were collected using Internal Review Board-approved protocols and de-identified to ensure patient confidentiality. In the TCGA dataset, each sample represents a unique case [26]. Principal Component Analysis was based on the expression of angiogenic-related genes. The first two components expressed 25% of total variability. A PLS model was then used to validate the discrimination ability of the CSF3 expression level. Patients were divided into two groups based on the CSF3 expression median (lower or higher than median). A PLS model was estimated with these two groups of CSF3 expression levels as the response factor. A 10-fold cross-validation procedure was then used to validate the predictive power of the model and led to 75% of correct classifications.

We also used another dataset, namely expression data available at the NCBI Geo datasets, accession number GSE72951, generated using Illumina HumanHT-12 WG-DASL V4.0 R2 expression BeadChip platform. Data were obtained from 115 patients included in the BELOB trial, which included glioblastoma-bearing patients at recurrence (35 treated with bevacizumab, 37 treated with lomustine and 43 with both) [27, 28].Focusing on patients treated with lomustine or lomustine+bevacizumab, the association between treatment efficacy and CSF3 expression was determined in the BELOB cohort. Expression data were provided using arbitrary units. Patients were separated into two groups using the best CSF3 expression cutoff using Cutoff Finder Software.

By using qPCR, we tested CSF3 expression in a series of 12 untreated GBM and tested the correlation between CFS3 expression and neutrophil count before surgery.

## RESULTS

### Exploratory cohort

#### Patients

Two hundred and sixty-five patients with GBM consecutively treated with the radiochemotherapy for GBM recommended since 2006 were included in this cohort. The clinical characteristics are summarized in (Supplementary Table 1, available online only). Twenty-two patients (8.3%) did not initially receive radiotherapy due to their large tumor size and received bevacizumab with chemotherapy as the first-line treatment. At recurrence, 28 (13.9%) were treated with local therapy (20 with surgery, 7 with stereotaxic radiotherapy and one with both) and 172 patients were treated with chemotherapy. One hundred and fifty-nine patients received a bevacizumab-based regimen, while 106 patients did not receive bevacizumab (Supplementary Table 2, available online only).

#### Clinical outcome

Median follow-up for this cohort was 51.5 months (range, 2.2-93.2 months); 245 patients died during follow up. The median OS in the whole population was 15.9 months (95% CI: 13.9-17.3). The median OS was improved in patients that received bevacizumab (18.7 months (95% CI: 16.8-21.0) *versus* 11.3 months (95% CI: 9.4-13.6) (*p* = 0.0014) (Supplementary Figure 1, available online only). We determined the best cut-off for neutrophil count at baseline to predict overall survival using Cutoff Finder Software (Supplementary Figure 2, available online only). The best cut-off was estimated at 5720/mm3, but as there was only a slight difference in the hazard ratio for a cut-off of 5720/mm3 and that of 6000/mm3, we chose to set the cut-off at 6000/mm3, which is also more clinically relevant. A high neutrophil count was strongly associated with poorer survival in the whole cohort (13.8 months (95% CI: 11.9-15.7) *versus* 18.6 months (95% CI: 15.9-21.6) (*p* = 0.0032) (Supplementary Figure 1, available online only)). In univariate and multivariate analysis, age, complete resection, Karnofsky performance status, baseline neutrophil count and treatment with bevacizumab were significantly associated with better OS (Table 1). Bootstrapped HR were more similar than crude HR (Table 1).

**Table 1:** Uni and multivariate analysis (Cox regression) for factors associated with OS

	Univariate HR	95% CI	*p*	Multivariate HR	95% CI	*p*	HR after bootstrap
**Age** ** <59** ** ≥59**	1 1.7	1.3-2.2	<0.0001	1 1.7	1.2-2.3	0.0004	1 1.7
**Sex** ** Female** ** male**	1 1.1	0.8-1.4	0.6				
**Diagnosis:** ** Biopsy** ** Surgery**	1 0.7	0.5-0.9	0.002	1 0.7	0.5-0.9	<0.0001	1 0.7
**Karnofsky status** ** <70** ** ≥70**	1 0.3	0.2-0.5	<0.0001	1 0.3	0.2-0.5	<0.0001	1 0.3
**Neoadjuvant chemotherapy** ** No** ** Yes**	1 0.8	0.8-1.5	0.2	1 0.9	0.6-1.6	0.18	1 0.9
**Bevacizumab use** ** No** ** Yes**	1 0.6	0.5-0.8	0.0015	1 0.7	0.5-0.9	0.008	1 0.7
**Neutrophil count** **<6000/mm^3^** ** >6000/mm^3^**	1 1.2	1.2-2	0.0035	1 1.6	1.1-2.1	0.005	1 1.7
**Lymphocyte count** ** <median** ** >median**	1 0.9	0.7-1.2	0.53				
**Monocyte count** ** <median** ** >median**	1 0.9	0.7-1.3	0.85				
**Hemoglobin value** ** <median** ** >median**	1 1.1	0.8-1.4	0.71				

**Figure 1:**
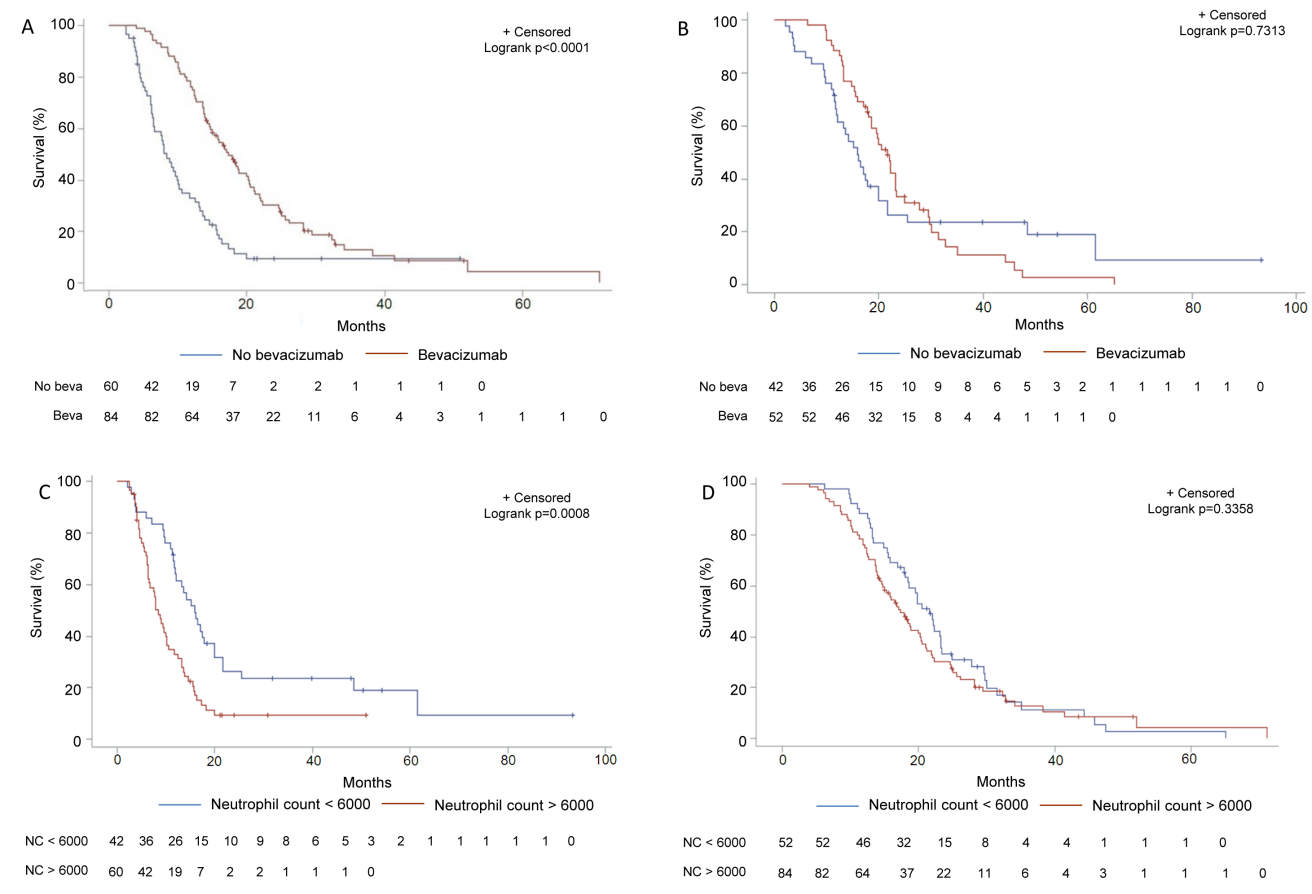
Subgroup analysis of survival in function of bevacizumab usage and neutrophil count in the training set **A.** Kaplan-Meier estimates of overall survival in patients treated or not with bevacizumab in the subgroup of patients with a high (≥6000/mm3) neutrophil count at baseline in the training cohort. **B.** Kaplan-Meier estimates of overall survival in patients treated or not with bevacizumab in the subgroup of patients with a low (< 6000/mm3) neutrophil count at baseline in the training cohort. **C.** Kaplan-Meier estimates of overall survival in patients with a high (>6000/mm3) *versus* low neutrophil count at baseline in the subgroup of patients treated with bevacizumab in the training cohort. **D.** Kaplan-Meier estimates of overall survival in patients with a high (>6000/mm3) *versus* low neutrophil count at baseline in the subgroup of patients treated without bevacizumab in the training cohort.

To determine whether the neutrophil count was only a prognostic factor or also a predictive factor of bevacizumab efficacy, we performed an interaction test between the neutrophil count at baseline and bevacizumab therapy using the previous multivariate Cox model. The interaction between the neutrophil count and bevacizumab therapy was significant in this multivariate model (Table 2). In the subgroup of patients with a high neutrophil count at baseline, bevacizumab therapy improved OS: 17.3 months (95% CI 14.6-20.4) *versus* 8.4 months (95% CI: 6.4-10.3), (*p* < 0.0001)). In the group of patients with a low neutrophil count, OS was not significantly improved in those treated with bevacizumab: 21.6 months (95% CI: 18.0-23.3) *versus* 15.9 months (95% CI: 12.0-19.9), (*p* = 0.7313))(Figure 1A and 1B). The neutrophil count remained a factor of a poor prognosis only in patients who did not receive bevacizumab, with OS of (8.4 months (95% CI 6.4-10.3) *versus* 15.9 months (95% CI 12.0-19.9) for patients with a high and low neutrophil count, respectively (*p* = 0.0008)) (Figure 1C). For patients treated with bevacizumab, the neutrophil count lost its prognostic ability (17.3 months (95% CI: 14.6-20.4) *versus* 21.6 months (95% CI: 18.0-23.3)) for patients with a high and low neutrophil count, respectively (*p* = 0.3358) (Figure 1D), suggesting that bevacizumab was able to counterbalance the deleterious effect of a high neutrophil count. To assess the stability of the neutrophil count to predict survival, we determined whether the neutrophil count at recurrence could predict bevacizumab efficacy. Using univariate and multivariate Cox models, we found that the neutrophil count at recurrence also predicted survival (Supplementary Table 3, available online only), and predicted bevacizumab efficacy (Supplementary Figure 3, available online only).

**Table 2:** multivariate analysis (Cox regression) for factors associated with OS with bevacizumab/neutrophil interaction

	multivariate HR	95% CI	*p*
**Age** ** <59** ** ≥59**	1 1.7	1.3-2.3	0.8
**Diagnosis:** **Biopsy** **Surgery**	1 0.7	0.5-0.97	0.035
**Karnofsky status** ** <70** ** ≥70**	1 0.4	0.2-0.6	<0.0001
**Neoadjuvant chemotherapy** ** No** ** Yes**	1 0.8	0.5-1.6	0.6
**Bevacizumab Neutrophil interaction**			0.02

### Validation cohort: the TEMAVIR cohort

**Figure 2:**
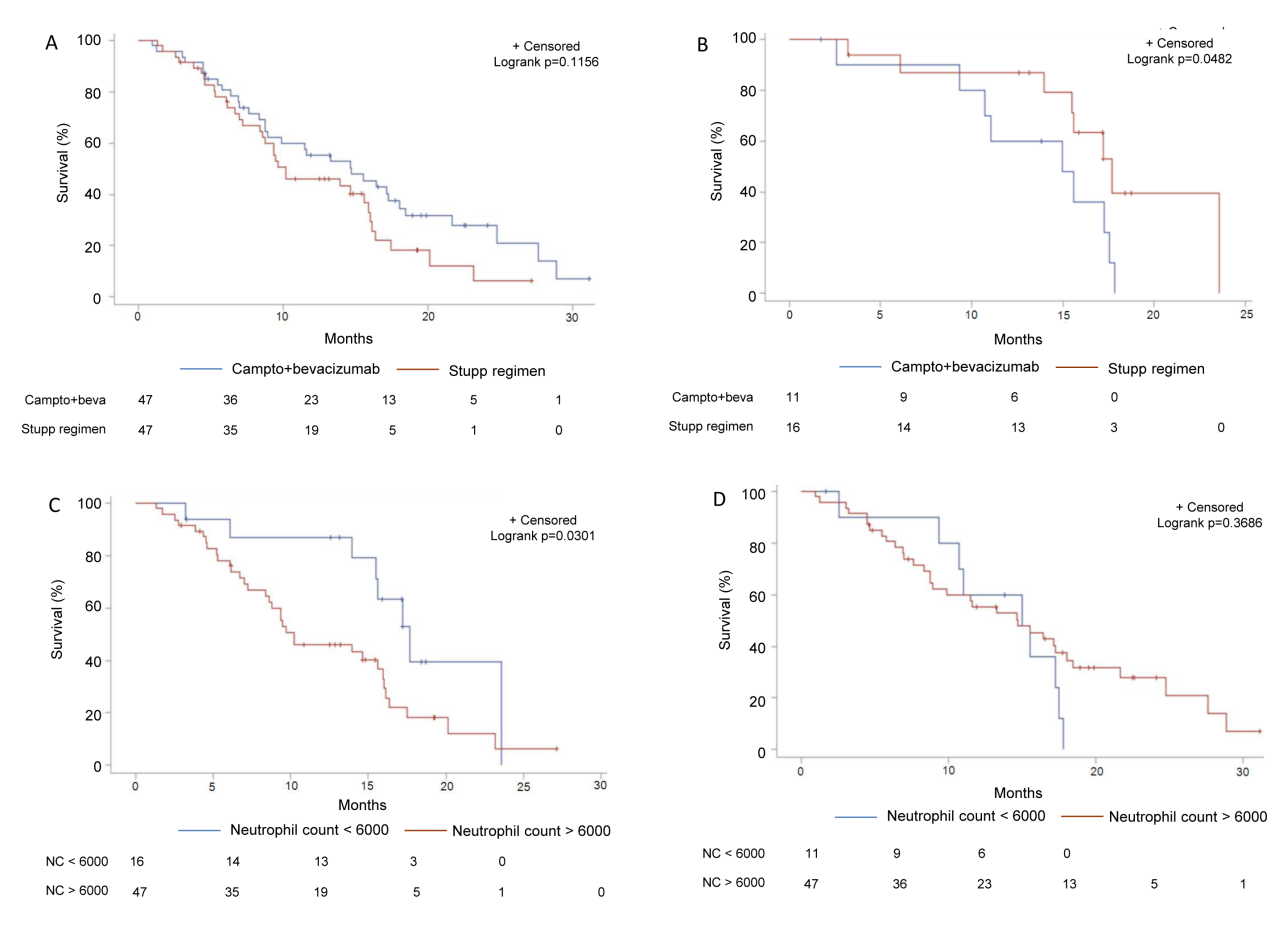
Subgroup analysis of survival in function of bevacizumab usage and neutrophil count in the validation set **A.** Kaplan-Meier estimates of overall survival in patients treated in the experimental arm with bevacizumab or in the standard arm without bevacizumab in the subgroup of patients with a high (≥6000/mm3) neutrophil count at baseline in the TEMAVIR cohort. **B.** Kaplan-Meier estimates of overall survival in patients treated in the experimental arm with bevacizumab or in the standard arm without bevacizumab in the subgroup of patients with a low (< 6000/mm3) neutrophil count at baseline in the TEMAVIR cohort. **C.** Kaplan-Meier estimates of overall survival in patients with a high (>6000/mm3) *versus* low neutrophil count at baseline in the subgroup of patients treated with bevacizumab in the experimental arm of the TEMAVIR cohort (bevacizumab use). **D.** Kaplan-Meier estimates of overall survival in patients with a high (>6000/mm3) *versus* low neutrophil count at baseline in the subgroup of patients treated with bevacizumab in the standard arm of the TEMAVIR cohort (no bevacizumab use).

The TEMAVIR clinical trial included 120 patients with grade IV unresectable GBM. In the experimental arm, 60 patients were treated with neoadjuvant bevacizumab and irinotecan for 2 months before radiochemotherapy. In the control arm, 60 patients were treated with the radiochemotherapy regimen. The clinical characteristics have been reported previously [24]. Both arms gave similar results in terms of PFS and OS [24]. The neutrophil count at baseline was not different between the two groups (mean baseline neutrophil count: 10 746.89 per mm3 (SD = 14040.43) in the experimental arm *vs* 8 471.83 per mm3 (SD = 3384.07) in the control arm (*p* = 0.3178). Using the previously defined cut-off, a high neutrophil count at baseline tended to be associated with a poorer prognosis but did not reach significance probably because of a lack of statistical power due to the small number of patients (median OS 13.3 months (95%CI: 9.3-5.9) *versus* 17.2 months (95%CI: 14.0-17.7), p = 0.368; not shown). The interaction between the neutrophil count and bevacizumab therapy was significant in the univariate Cox model for OS (*p* = 0.0294). In the group of patients with a high neutrophil count at baseline, we also observed that bevacizumab improved OS, though the difference was only borderline significant (14.7 months (95% CI: 8.8-18.0) v*ersus* 10.2 months (95% CI: 8.4-15.9), log rank test *p* = 0.1156 for patients from experimental versus control arm) (Figure 2A). In contrast, in the group of patients with a low neutrophil count, treatment with bevacizumab was deleterious compared with radiochemotherapy (15.0 months (95% CI: 2.6-17.5) *ersus* 17.7 months (95% CI: 14.0-23.6), log rank test; *p* = 0.0482) (Figure 2B). For patients in the control arm (radiochemotherapy), a high neutrophil count was associated with a poor prognosis: (10.2 months (95% CI: 8.4-15.9) *versus* 17.7 months (95% CI: 14.0-23.6), log rank test; *p* = 0.0301) (Figure 2C). For patients in the experimental arm (bevacizumab), the prognostic value of the neutrophil count disappeared (14.7 months (95% CI: 8.8-18.0) *versus* 15.0 months (95% CI: 2.6-17.5); for patients with a high vs low neutrophil count, respectively, log rank test; *p* = 0.3686) (Figure 2D). This seems to confirm that bevacizumab was also able to compensate for the deleterious effect of a high neutrophil count in this independent cohort.

### Prognostic value of G-CSF in GBM

**Figure 3:**
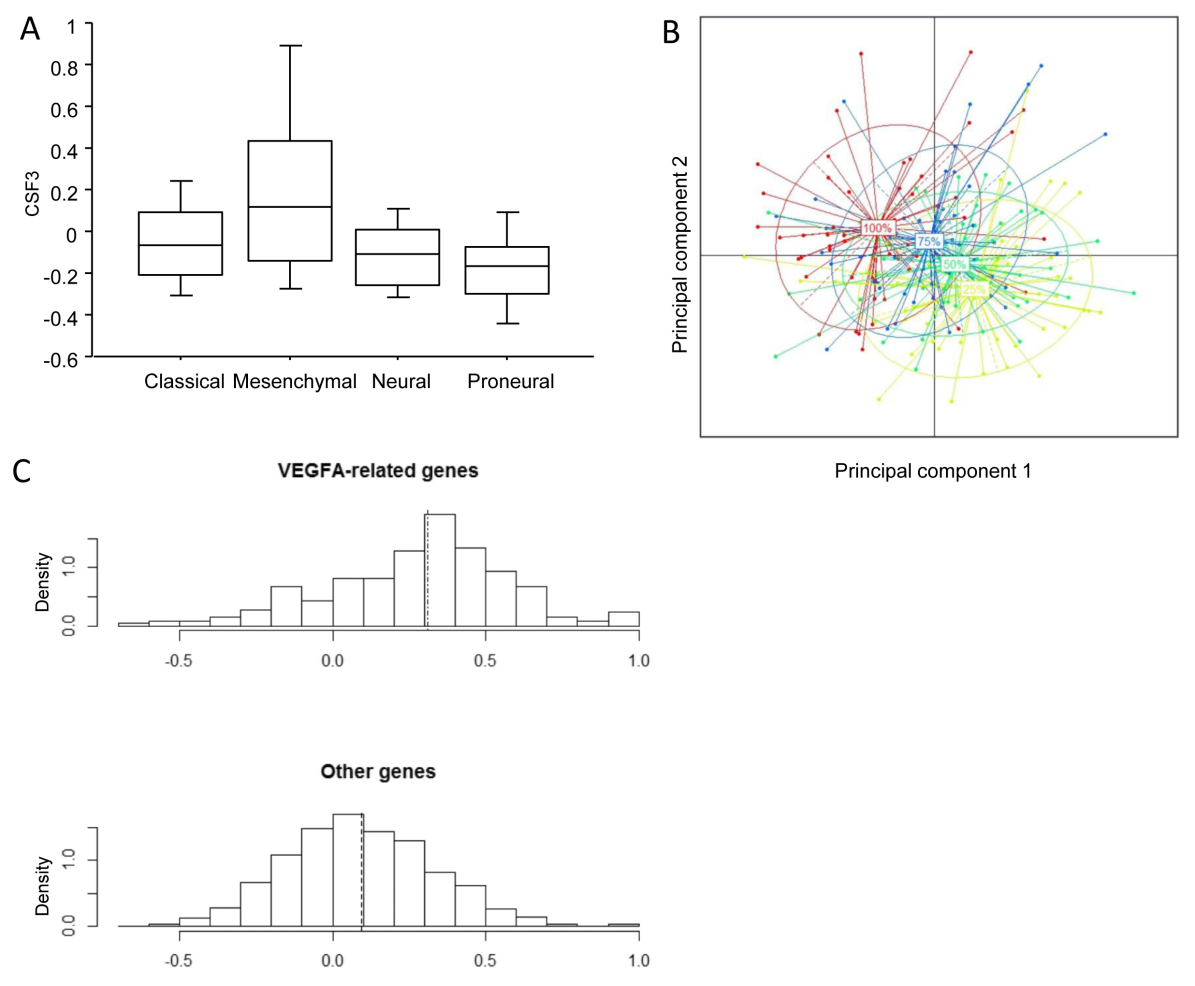
Bioinformatic analysis of relation of CSF3 expression with molecular subtype of glioblastoma and angiogenic process **A.** Expression of *CSF3* mRNA in the 202 glioblastoma patients of the TCGA classified according to the molecular classification of glioblastoma. **B.** First plane of the Principal Component realized on the 202 glioblastoma patients using angiogenic genes. Each point on the graph represents one patient. The colored ellipses represent the inertia in each group delimited by the quartiles of the CSF3 gene expression. Groups were established as follows: “25%” corresponds to patients with CSF3 expression below the first quartile; “50%” corresponds to patients with CSF3 expression between the first and the second quartiles; “75%” corresponds to patients with CSF3 expression between the second and the third quartiles; “100%” corresponds to patients with CSF3 expression above the third quartile. The percentage of variance reflected by horizontal axe is 16% and 10% by vertical axis. **C.** Top: Histogram of the correlations observed between CSF3-related genes and VEGFA-related genes. Bottom: Histogram of the correlations observed between CSF3-related genes and VEGFA-non related genes. The mean of the distribution is visualized through the vertical dashed lines.

To gain deeper understanding of the link between the effect of bevacizumab and the neutrophil count at baseline, we wondered whether GBM could secrete a growth factor for neutrophils such as Granulocyte-Colony Stimulating Factor (G-CSF). Importantly, in a series of 12 GBM, CSF3 (the gene coding for G-CSF) expression in the tumor bed correlated strongly with the neutrophil count (r^²^ = 0.36; *p* = 0.04). Using public transcriptomic data from The Cancer Genome Atlas [26] for 202 patients suffering from GBM, we found that *CSF3* was overexpressed mostly in mesenchymal glioblastoma (Figure 3A). Principal component analysis (PCA) based on angiogenesis-related genes (Supplementary Table 4A, available online only) showed that the main part of the variability was linked to CSF3 expression (Figure 3B). In addition, we validated our observation through a Partial least square (PLS) model by showing that expression of angiogenic genes could be used to discriminate patients according to their CSF3 expression with an accuracy of 75% obtained by 10-fold cross-validation (Supplementary Figure 4, available online only). Together, these data underline that *CSF3* expression is related to a specific type of angiogenic process. Moreover, we observed that *VEGFA*-related angiogenic genes correlated more strongly with *CSF3*-related genes than with *VEGFA* independent angiogenic genes. This suggests that CSF3 high-expressing tumors were more strongly associated with *VEGFA*-dependent angiogenesis than were *CSF3* low-expressing tumors (Figure 3C) (Supplementary Table 4B and C, available online only)

Using transcriptomic data from the BELOB phase II trial, which compared lomustine *versus* lomustine plus bevacizumab in recurrent glioblastoma, we confirmed that *CSF3* expression was overexpressed in mesenchymal tumors and correlated with *VEGFA* expression (see eFigure 5 in the Supplement). In the group of patients with a high *CSF3* level, lomustine plus bevacizumab was associated with better OS (12.3 months (95% CI: 8.4-14.6) *versus* 7.6 months (95% CI: 5.8-10.3), log rank test *p* = 0.0212) (see eFigure 6 in the Supplement). In contrast, in the group of patient with low a *CSF3* level there was a non-significant trend towards a deleterious effect of lomustine plus bevacizumab (5.9 months (95% CI: 4.4-10.8) *versus* 13.9 months (95% CI: 2.9-19.6), log rank test; *p* = 0.35) (see eFigure 6 in the Supplement). For patients treated with lomustine alone, a high *CSF3* was associated with poor OS: (7.6 months (95% CI: 5.5-10.2) versus 13.2 months (95% CI: 11-19.5), log rank test; *p* = 0.0442) (see eFigure 6 in the Supplement). For patients treated with lomustine and bevacizumab, the prognostic value of CFS3 disappeared (12.3 months (95% CI: 9.5-14.6) *versus* 5.8 months (95% CI: 4.4-10.8); for patients with a high vs low CSF3, respectively, log rank test; *p* = 0.28) (see eFigure 6 in the Supplement). Together, these data demonstrate that tumor *CSF3* expression, like the neutrophil count, can predict bevacizumab efficacy.

## DISCUSSION

Together, these data underscore the capacity of a high peripheral neutrophil count to predict bevacizumab efficacy in GBM both at recurrence and in the neoadjuvant setting. While the neutrophil count was strongly associated with the benefit of bevacizumab in terms of OS in the first cohort, data were only borderline significant in the TEMAVIR cohort. The absence of statistical significance was probably due to the lack of power given the small number of patients. Besides, 25% of patients in the control arm (radiochemotherapy) received bevacizumab at recurrence, which could have strongly biased survival results.

High-grade gliomas secrete large amounts of vascular endothelial growth factor (VEGF), which acts in a paracrine manner to promote endothelial cell proliferation, survival, and migration [29]. Inhibiting VEGF is an efficient anticancer therapy in many cancer types [30, 31, 32] and bevacizumab is currently used for the treatment of recurrent glioblastoma [33,9, 28, 34]. As bevacizumab targets VEGF-A, it is plausible that tumors with high VEGF-A expression and whose angiogenesis is mainly dependent on VEGF-A are more likely to respond to bevacizumab. GBM have often been reported to produce G-CSF [35–37]. In addition, G-CSF induces paracrine and autocrine activation of GBM cells, thus rendering GBM cells resistant to apoptosis and able to promote pro-angiogenic pathways *via* the increased expression of VEGF-A [38–40]. In addition, G-CSF could also induce VEGF-A production by microenvironmental microglia cells [41]. These data may explain why high *CSF3* expression in GBM is associated with the response to bevacizumab. In contrast, intratumoral neutrophil infiltration was previously associated with resistance to bevacizumab [42]. We could suspect that the peripheral neutrophil count does not directly affect bevacizumab efficacy but is rather a surrogate marker of high *CSF3* expression, as demonstrated by the correlation between *CSF3* expression and the neutrophil count.

Recently, two randomized phase III trials - AVAglio (Avastin in Glioblastoma) and RTOG-0825 (Radiation Therapy Oncology Group 0825) - investigated the addition of bevacizumab to standard-of-care therapy in newly diagnosed GBM [13, 14]. Both studies reported longer median PFS with bevacizumab *versus* placebo (AVAglio: 10.6 *v* 6.2 months; hazard ratio [HR], 0.64; RTOG 0825: 10.7 *v* 7.3 months; HR, 0.79) [13, 14]. The increased PFS did not translate into an expected OS benefit in the intent-to-treat population in either study, suggesting that bevacizumab is not effective and may even be deleterious in certain patients with glioblastoma. The failure of bevacizumab to improve overall survival in a global population of glioblastoma was also underlined at recurrence in the recent EORTC 26101 trial, which tested the effect of adding bevacizumab to the lomustine regimen in recurrent glioblastoma [15]. The molecular profiles established in the RTOG and AVAglio studies raised the hypothesis that mesenchymal or proneuronal phenotypes were associated with the response to bevacizumab as the first-line therapy [43]. These data contradict our results. These discrepancies could be due to differences in inclusion criteria and treatments in the different cohorts. In particular, in AVAGLIO and RTOG 0825, patients were treated with first-line bevacizumab. In addition, many patients received bevacizumab in the second-line, which could have affected the results. In our retrospective cohort, patients were given bevacizumab mainly as the second-line treatment. The patients included in the TEMAVIR clinical trial were suffering from unresectable GBM, and could not have been included in AVAGLIO and RTOG0825. However, due to the simplicity of our biological testing, a retrospective study of the effect of the neutrophil count in AVAGLIO and RTOG0825 should be performed. In addition, the EORTC phase III trial (NCT01290939), which compared lomustine *versus* bevacizumab plus lomustine in the second-line, was close to ours from a clinical viewpoint, and it could be interesting to evaluate the predictive role of the neutrophil count in these studies as a second external validation cohort to confirm our results.

In conclusion, we propose that the neutrophil count at baseline could be used as a surrogate predictor of bevacizumab efficacy in GBM. However, this use of neutrophil count as a predictor of efficacy of bevacizumab is probably tumor specific. This could be due to conflicting results found in the literature in lung and colorectal cancer [44, 45]. The particularity of GBM may be related to the direct ability of tumor cells to produce G-CSF and thus promote the expansion of neutrophils. These G-CSF secreting GBM are more proangiogenic, thus providing a biological rationale to explain their sensitivity to bevacizumab. Our results could be quickly validated using AVAGLIO, RTOG0825 and EORTC (NCT01290939) data.

## SUPPLEMENTARY MATERIAL


