# Supplementary material for: Blood baseline neutrophil count predicts bevacizumab efficacy in glioblastoma
Source: Oncotarget. 2016 Jul 28;7(43):70948–58. doi: 10.18632/oncotarget.10898 (PMC5342600; doi:10.18632/oncotarget.10898)
Supplement: Supplementary file 1 [file oncotarget-07-70948-s001.pdf]

Blood baseline neutrophil count predicts bevacizumab efficacy in glioblastoma

Supplementary Material

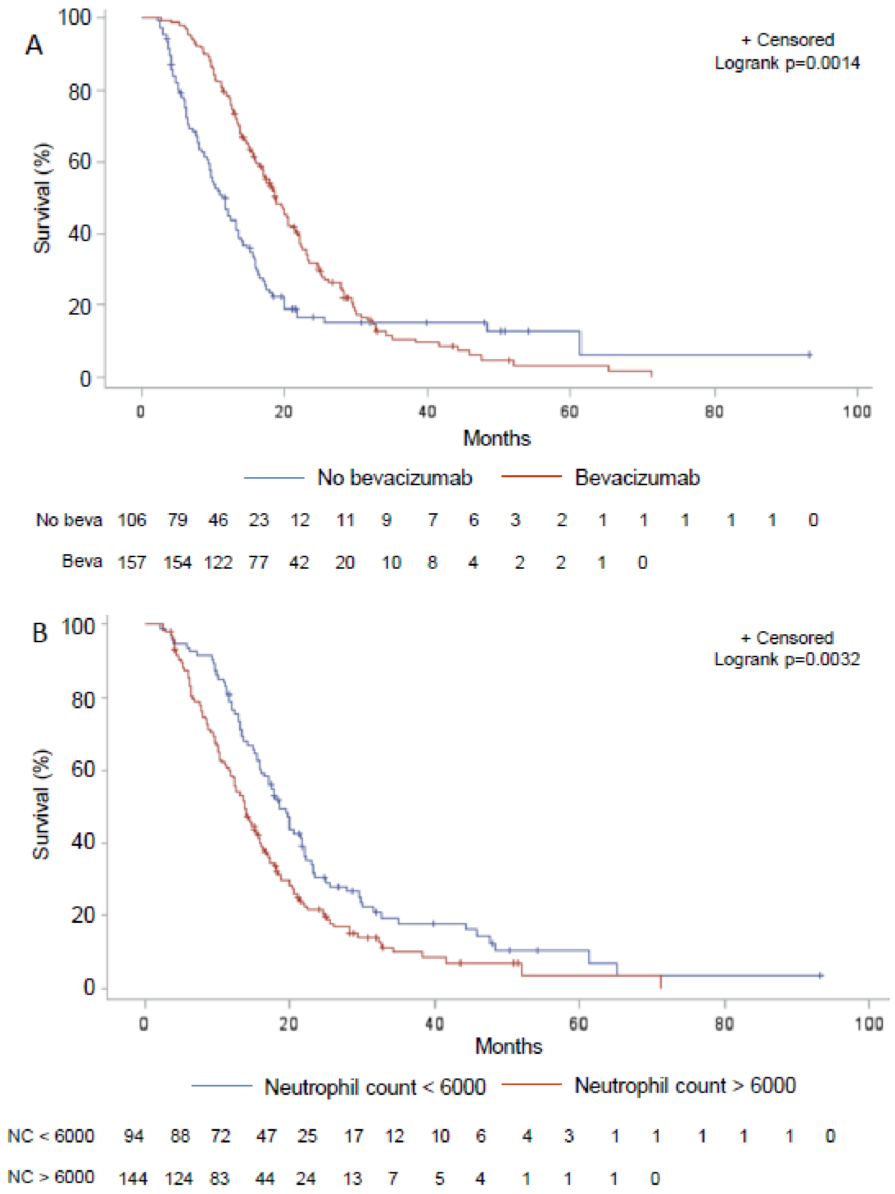

Supp. Figure 1: Estimation of survival in function of bevacizumab treatment of neutrophil count

A. Kaplan–Meier estimates of overall survival in patients treated or not with bevacizumab in the training cohort.

B. Kaplan–Meier estimates of overall survival in patients with a high (>6000/mm3) versus low neutrophil count at baseline in the training cohort

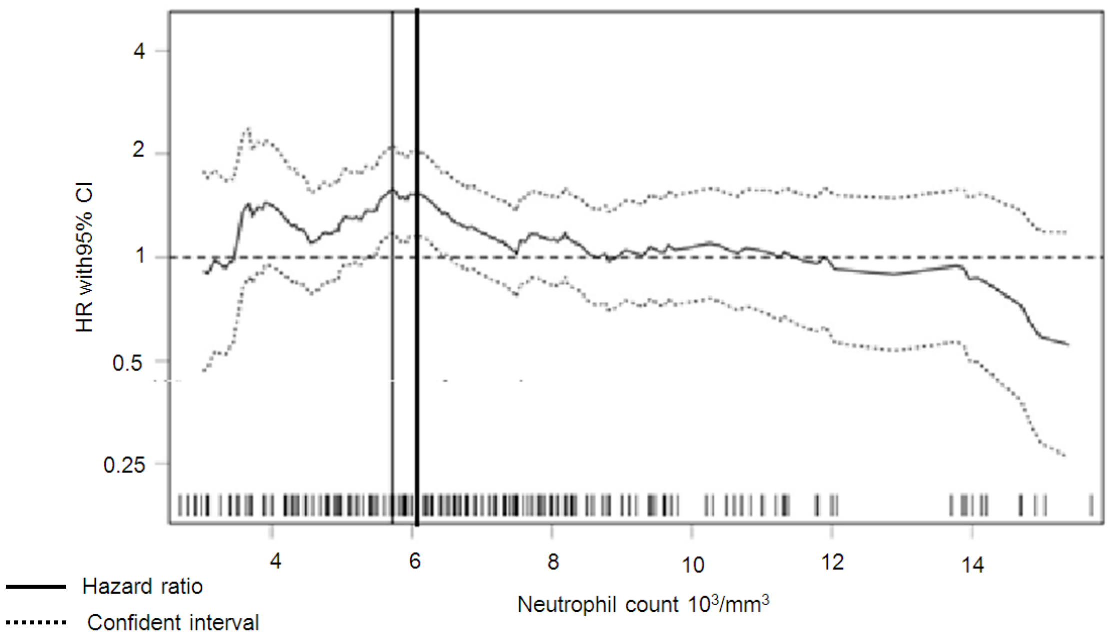

Supp. Figure 2: Determination of optimal cutoff of neutrophil.

For each possible cutoff, neutrophil count was correlated with overall survival. The hazard ratio (HR) including 95% CI is plotted in dependence of the cutoff. A vertical line designates the dichotomization showing the most significant correlation with survival. The distribution of the neutrophil count in the 265 patients is shown as a rug plot at the bottom of the figures.

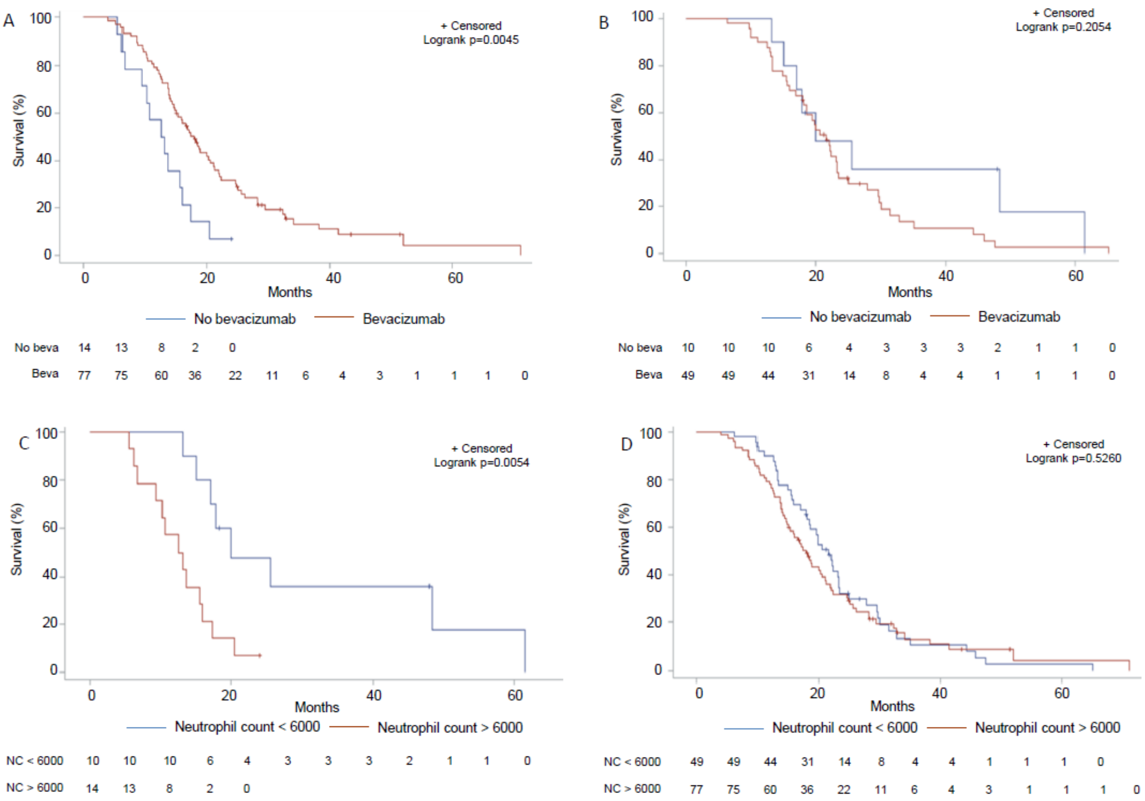

Supp. Figure 3: Subgroup analysis of survival in function of bevacizumab usage at recurrence and neutrophil count using neutrophil count determine at recurrence

A. Kaplan–Meier estimates of overall survival in patients treated or not with bevacizumab in the subgroup of patients with a high (≥6000/mm3) neutrophil count at recurrence.
B. Kaplan–Meier estimates of overall survival in patients treated or not with bevacizumab in the subgroup of patients with a low (<6000/mm3) neutrophil count at recurrence.
C. Kaplan–Meier estimates of overall survival in patients with a high (>6000/mm3) versus low neutrophil count at recurrence in the subgroup of patients treated with chemotherapy with bevacizumab in the training cohort.
D. Kaplan–Meier estimates of overall survival in patients with a high (>6000/mm3) versus low neutrophil count at recurrence in the subgroup of patients treated with chemotherapy without bevacizumab

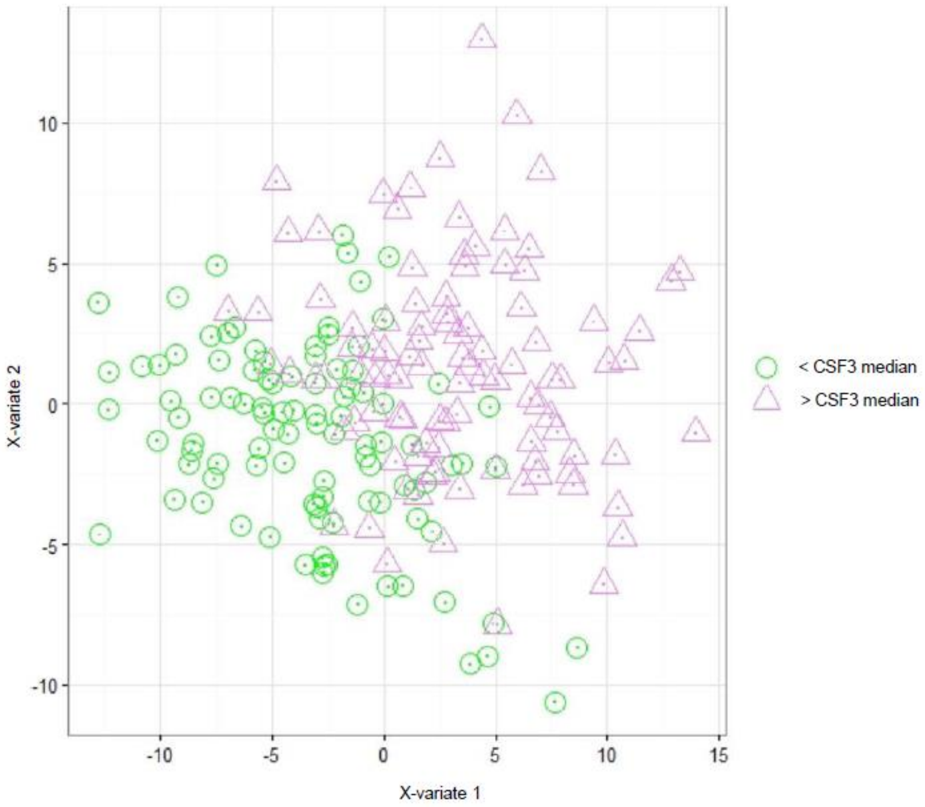

Supp. Figure 4: Partial least square regression model using CSF3 expression to predict the type of angiogenic process

Patients were divided into two groups based on the CSF3 expression median (lower or higher than median). A PLS model was estimated with these 2 groups of CSF3 expression levels as response factor. A 10-fold cross-validation procedure was then used to validate the predictive power of the model and led to 75% of correct classifications

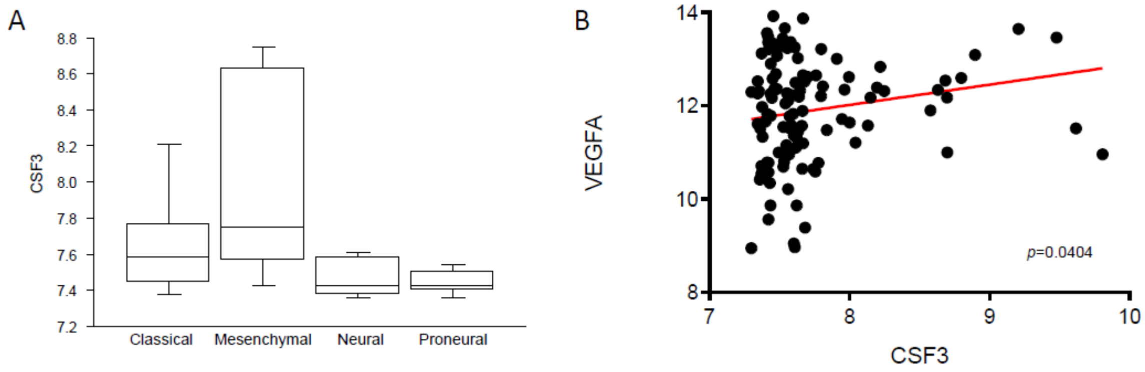

Supp. Figure 5: Boinformatic analysis of relation of CSF3 expression with molecular subtype and aVEGFA expression.

A. Expression of *CSF3* mRNA in the 115 glioblastoma patients treated in BELOB trial and classified according to the molecular classification of glioblastoma
B. Correlation between *CSF3* and *VEGFA* mRNA in the 115 glioblastoma patients treated in BELOB trial

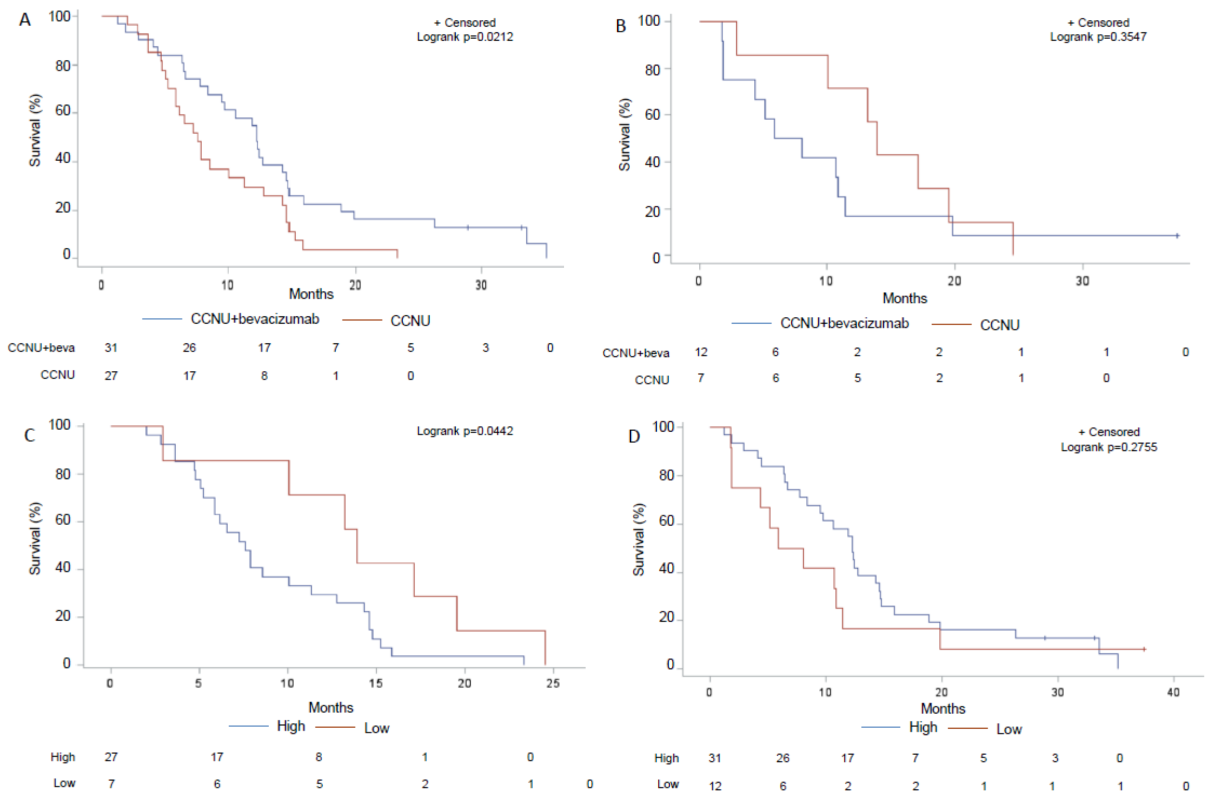

Supp. Figure 6: Subgroup analysis of survival in function of bevacizumab usage and CSF3 expression in BELOB study

A. Kaplan–Meier estimates of overall survival in patients treated with CCNU alone or with bevacizumab in the subgroup of patients with a high *CSF3* expression in tumor sample analysis.
B. Kaplan–Meier estimates of overall survival in patients treated with CCNU alone or with bevacizumab in the subgroup of patients with a low *CSF3* expression in tumor sample analysis.
C. Kaplan–Meier estimates of overall survival in patients with a high versus low *CSF3* expression in tumor sample analysis in the subgroup of patients treated with CCNU alone.
D. Kaplan–Meier estimates of overall survival in patients with a high versus low *CSF3* expression in tumor sample analysis in the subgroup of patients treated with CCNU and bevacizumab

Supp Table 1 : Patients and tumor characteristics (N=265)

		N (%)
**Sex**		
	male	159 (60%)
	female	106 (40%)
**Age (years)**	mean (SD)	59.8 (11.6)
	median (min-max)	61 [20-86]
**Neutrophil count at baseline (cell/mm^3)**		
	mean (SD)	7400 (3600)
	median (min-max)	6700 [800-22100]
**Karnofsky status (%)**		
	30	2 (0.8%)
	40	5 (1.9%)
	50	8 (3.1%)
	60	10 (3.9%)
	70	31 (12%)
	80	13 (5%)
	90	111 (42.9%)
	100	79 (30.5%)
	missing	6
**Diagnosis**		
	Biopsy	104 (39.5%)
	surgery	159 (60.5%)
	missing	2
**Relapse**		
	No	15 (6.9%)
	Yes	201 (93.1%)
	missing	49

Supp Table 2 : treatment characteristics (N=265)

				N (%)
**Neoadjuvant chemotherapy**				
	Yes			22 (8.3%)
	No			243 (91.7%)
**Adjuvant temozolomide**				59 (22.3%)
	Yes			206 (77.7%)
	No			
**Relapse therapy (n=201)**				
	**Local therapy**			28 (13.9%)
		Surgery	20	
		radiotherapy	7	
		both	1	
	**chemotherapy**			172 (85.6%)
	**Palliative care**			26 (12.9%)
**Bevacizumab use**				
	No			106 (40%)
	Yes (including)			159 (60%)
	Neoadjuvant		17	
	Concomitant with radiotherapy		23	
	adjuvant		22	
	At recurrence		148	

Supp Table 3: multivariate analysis (Cox regression) for factors associated with OS using neutrophil at recurrence

	multivariate HR	95% CI	p
**Age**			**0,0055**
**< 59**	1		
**≥ 59**	1,6	1,2-2,3	
**Diagnosis**			**0,0674**
**stereotaxic biopsy**	1		
**surgery**	0,7	0,5-1	
**Karnofsky status**			**0,0132**
**≤ 70**	1		
**> 70**	0,5	0,2-0,8	
**Neochemotherapy**			**0,126**
**No**	1		
**Yes**	0,6	0,3-1,2	
**Bevacizumab use at recurrence**			
**No**	1		**0.416**
**Yes**	0,8	0,5-1,3	
**Neutrophil count at recurrence**			
**< 6000**	1		**0.0071**
**≥ 6000**	1,6	1,1-2,3	

Supplementary Table 4.
